# Brain Volumetric Analysis Using Artificial Intelligence Software in Premanifest Huntington’s Disease Individuals from a Colombian Caribbean Population

**DOI:** 10.3390/biomedicines12102166

**Published:** 2024-09-24

**Authors:** Margarita R. Ríos-Anillo, Mostapha Ahmad, Johan E. Acosta-López, Martha L. Cervantes-Henríquez, Maria C. Henao-Castaño, Maria T. Morales-Moreno, Fabián Espitia-Almeida, José Vargas-Manotas, Cristian Sánchez-Barros, David A. Pineda, Manuel Sánchez-Rojas

**Affiliations:** 1 Facultad de Ciencias de la Salud, Centro de Investigaciones en Ciencias de la Vida, Universidad Simón Bolívar, Barranquilla 080005, Colombia; mostapha.ahmad@unisimon.edu.co (M.A.); jose.vargas@unisimon.edu.co (J.V.-M.); sanchezr@unisimonbolivar.edu.co (M.S.-R.); 2 Médico Residente de Neurología, Facultad de Ciencias de la Salud, Centro de Investigaciones en Ciencias de la Vida, Universidad Simón Bolívar, Barranquilla 080005, Colombia; maria.henao2@unisimon.edu.co (M.C.H.-C.); maria.morales5@unisimon.edu.co (M.T.M.-M.); 3 Facultad de Ciencias Jurídicas y Sociales, Centro de Investigaciones en Ciencias de la Vida, Universidad Simón Bolívar, Barranquilla 080005, Colombia; johan.acosta@unisimon.edu.co (J.E.A.-L.); martha.cervantes@unisimon.edu.co (M.L.C.-H.); 4 Facultad de Ciencias Básicas y Biomédicas, Centro de Investigaciones en Ciencias de la Vida, Universidad Simón Bolívar, Barranquilla 080005, Colombia; fabian.espitia@unisimon.edu.co; 5 Departamento de Neurofisiología Clínica Palma de Mallorca, Hospital Juaneda Miramar, 07001 Palma, Spain; doctorcristiansanchez@gmail.com; 6 Grupo Neuropsicología y Conducta, Universidad de San Buenaventura, Medellín 050021, Colombia; david.pineda1@udea.edu.co; 7 Grupo de Neurociencias de Antioquia, Universidad de Antioquia, Medellín 050010, Colombia

**Keywords:** artificial intelligence, Huntington’s disease, magnetic resonance imaging, neuroimaging, structural MRI

## Abstract

**Background and objectives:** The premanifest phase of Huntington’s disease (HD) is characterized by the absence of motor symptoms and exhibits structural changes in imaging that precede clinical manifestation. This study aimed to analyze volumetric changes identified through brain magnetic resonance imaging (MRI) processed using artificial intelligence (AI) software in premanifest HD individuals, focusing on the relationship between CAG triplet expansion and structural biomarkers. **Methods:** The study included 36 individuals descending from families affected by HD in the Department of Atlántico. Sociodemographic data were collected, followed by peripheral blood sampling to extract genomic DNA for quantifying CAG trinucleotide repeats in the Huntingtin gene. Brain volumes were evaluated using AI software (Entelai/IMEXHS, v4.3.4) based on MRI volumetric images. Correlations between brain volumes and variables such as age, sex, and disease status were determined. All analyses were conducted using SPSS (v. IBM SPSS Statistics 26), with significance set at *p* < 0.05. **Results:** The analysis of brain volumes according to CAG repeat expansion shows that individuals with ≥40 repeats evidence significant increases in cerebrospinal fluid (CSF) volume and subcortical structures such as the amygdalae and left caudate nucleus, along with marked reductions in cerebral white matter, the cerebellum, brainstem, and left pallidum. In contrast, those with <40 repeats show minimal or moderate volumetric changes, primarily in white matter and CSF. **Conclusions:** These findings suggest that CAG expansion selectively impacts key brain regions, potentially influencing the progression of Huntington’s disease, and that AI in neuroimaging could identify structural biomarkers long before clinical symptoms appear.

## 1. Introduction

Huntington’s disease (HD), an autosomal dominant monogenic neurodegenerative disease, was first described in 1872 by George Huntington, who reported an inherited choreiform disorder [1] resulting from the expansion of cytosine, adenine, and guanine (CAG) tract in the *IT15* gene (Huntingtin gene, *HTT*), located in the region 4p16.3. This protein encodes a polyglutamine, widely expressed in various tissues of the body and necessary for normal development [2]. One of these processes occurs during the 13th week of pregnancy, known as “interkinetic nuclear migration”, and is essential for developing corticostriatal projections [3]. 

Molecularly, normal polyglutamine differs from the mutated form in the length of the CAG repeats—those greater than 35 code for pathogenic polyglutamine. 

At the functional level, under normal conditions, this protein forms aggregates that could exert a neuroprotective function. The mutated polyglutamine also produces these same aggregates, which lead to dysfunction that affects multiple cellular pathways, including DNA replication and repair, transcription, splicing, and mitochondrial function, with the striatum and the cortex being the storage location. Even though all of these mechanisms have been described, they remain poorly understood [4].

HD manifests clinically with characteristics of motor dysfunction such as chorea, dystonia, bradykinesia, and rigidity, in addition to cognitive and psychiatric conditions [5]. Phenotypically, the number of CAG repeats that the carrier has translated into the possible age of onset of the disease and the severity of symptoms in the function of their quantity. It affects both sexes equally, and the risk of passing the gene to the next generation is 50% [6]. It generally manifests between 30 and 50 years of age, although an earlier form occurs before the age of 20 [7] and has been associated with expansion in CAG repeats greater than 60 [8].

The worldwide prevalence of HD is 2.71 per 100,000 inhabitants [9]. In Colombia, orphan diseases are defined as those that are chronically debilitating, serious, life-threatening, and with a prevalence of less than 1 per 5000 individuals. HD is endemic in a municipality located 40 km from the capital of the Department of Atlántico on the Colombian Caribbean coast with a total population of 17,487 inhabitants, according to the municipality’s official website (http://www.juandeacosta-atlantico.gov.co/, accessed on 12 January 2024). It stands out for being considered the second place with the highest number of patients affected by this disease worldwide. The estimated prevalence of HD in the Department of Atlántico is 0.2 per 10,000 inhabitants; however, in this municipality, it rises to 9.7 per 10,000 inhabitants [10].

Although the diagnosis of this disease is based on the onset of several types of abnormal movements (such as chorea, dystonia, fine motor impairment, dysarthria, dysphagia, and gait disorders) [8,11,12], the structural changes in the brain caused by volume loss, and defects in the corticstriatal network that cause dysfunction and degeneration of the striatum [3] can occur much earlier than motor symptoms [13]. They are a product of deposits of mutated *HTT*, which appear from very early stages of embryonic development. Studies have described these alterations in human and murine brain tissue from week 13 of pregnancy [3]. These tissues showed clear abnormalities in the developing cortex, including mislocalization of mutant *HTT* and junctional complex proteins, defects in neuroprogenitor cell polarity and differentiation, abnormal ciliogenesis, and changes in mitosis and cell cycle progression. However, the path to degeneration is complex and involves pathogenic and compensatory mechanisms, so the estimated period between these changes in neurodevelopment (and their subsequent alterations and motor manifestations) cannot be established [14]. 

The morphological changes brought about by this disease can be observed through neuroimaging techniques, such as structural magnetic resonance imaging (MRI) and brain volumetry, which allow for visualization and quantification of changes in the different regions of the brain [15]. Structural MRI has been widely used in HD investigation to determine volumetric changes in both manifest and premanifest patients [15]. Through this, different brain regions have been identified that have potential as biomarkers of progression in HD [16].

Until this study, these brain structural volumetric analyses used tools such as parametric statistical mapping and geometric progression models [17,18]. Currently, analysis using artificial intelligence (AI) is considered a pioneering, novel, and future-oriented tool in this field.

The advent of AI and the application of deep learning algorithms in neuroimaging have made it possible to detect complex and subtle patterns by training with extensive datasets of brain images specific to the Colombian population. With this, a precise analysis of the brain volumes of this population was conducted. These datasets were carefully curated and validated to ensure representativeness and relevance in the local context. Through this training, AI can identify relevant patterns and characteristics, especially in the premanifest HD population. During the volumetric process, AI performs automatic segmentations of specific brain structures with high precision, facilitating the quantification of volumetric changes. 

AI’s effectiveness in brain volumetry lies in its ability to rapidly process large datasets, enhance accuracy in identifying regions of interest, and ultimately serve as a valuable tool for early diagnosis and research in neurological diseases. This study proposes that the application of AI in neuroimaging could serve as an alternative method for identifying structural biomarkers long before patients present clinical symptoms, potentially up to 10 years earlier in premanifest patients. This capability is crucial for the early detection and intervention in HD, potentially altering the disease trajectory [19,20].

The integration of AI in neuroimaging is not only innovative but also supported by a growing body of literature that highlights its advantages in early disease detection and the analysis of complex datasets [21,22]. These studies underscore the transformative potential of AI in enhancing our understanding of HD and improving patient outcomes through earlier and more accurate diagnoses [19]. 

## 2. Materials and Methods 

### 2.1. Study Participants

The study included 36 individuals who were descendants of families affected by HD in the Department of Atlántico. Participation was voluntary, and participants had to meet specific criteria, including signing an informed consent. Individuals with other movement disorders or psychiatric history were excluded. Participants were part of an extensive molecular and genetic epidemiological evaluation of 291 individuals from the Juan de Acosta community [23,24]. To ensure a representative and unbiased sample, participants were selected based on a stratified random sampling technique, considering age, gender, and family history of HD. Any potential biases in selection were mitigated by applying inclusion and exclusion criteria consistently across the entire participant pool. Inclusion and exclusion criteria are listed in Table 1.

### 2.2. Clinical Assessment

#### 2.2.1. Diagnosis of Premanifestations of HD 

Family members with a confirmed case of HD underwent a structured neurological evaluation performed by two board-certified neurologists—one of whom is internationally certified in the application of the Unified Huntington’s Disease Rating Scale (UHDRS) [25]. To ensure the consistency and reliability of the assessments, all evaluations were conducted in a double-blind manner, where the evaluators were unaware of the participants’ genetic status during the neurological examination. Furthermore, a review committee was established to verify the accuracy and consistency of the results, ensuring that all evaluations adhered to the standardized procedures outlined. This evaluation included a structured interview, both with the patient and a close family member, about the presence or absence of typical motor symptoms of HD, such as speech alterations, choreic movements, TICs, tremors, dystonic movements, and changes in gait, using a Yes/No binary response format. Furthermore, the functionality of the participants was explored regarding their autonomy in work activities, financial management, domestic tasks, and basic self-care and hygiene activities, following the guidelines established by Myers (1988) [26].

Participants who reported no symptoms and proved to be fully functional and autonomous underwent a comprehensive neurological evaluation using the UHDRS standardized procedures. This included assessment of eye-tracking movements, saccades, speech, tongue protrusion, index finger and thumb tapping speed, hand pronation/supination, Luria motor organization, upper limb muscle tone, presence of chorea, dystonia, bradykinesia, gait analysis, tandem walking, and retropulsion evaluation. Only subjects who were classified with a confidence level of 0 (zero), that is, without symptoms, with complete autonomy, and no motor signs, were selected to participate in the study and subsequently underwent blood sample extraction for genotyping.

##### Blood Samples and Genomic DNA Extraction

Peripheral blood samples (5 mL) were taken from participants and stored at 4 °C until analysis. DNA was extracted using the Quiagen DNeasy Blood & Tissue kit (Hilden, Germany) to obtain a high purity product. The DNA was resuspended in ultrapure water and stored at −20 °C until further analysis, with its concentration and purity quantified using dsDNA HS Qubit™ 2.0 fluorometer assay kits. Quality control measures were implemented throughout the extraction process to ensure the integrity of the DNA samples. This included the use of duplicate samples and internal controls to monitor for potential contamination or degradation.

##### HTT CAG Repeat Quantification

The genomic DNA was sent to the iLab from the University of Arizona (Tucson, AZ, USA) for amplicon sequencing. This protocol allows for hundreds of samples to be sequenced in a single MiSeq run. Library preparation and MiSeq sequencing for genotyping were performed according to protocol. The sequence encoding the polyglutamine and polyproline tracts of the *HTT* gene was amplified from genomic DNA using MiSeq-compatible polymerase chain reaction (PCR) primers. After PCR, the products were purified and subjected to quality control before sequencing on the MiSeq platform. Stringent quality control procedures were applied throughout the sequencing process, including the use of positive and negative controls to validate the accuracy of the genotyping results. PCR primers were designed based on the TruSeq dual combinatorial design, with the addition of spacers between the binding site of the sequencing primer and the [23,27] locus-specific primer. Subsequently, 36 out of the 291 participants underwent brain MRI with volumetry.

##### MRI Volumetry

Brain magnetic resonance images were acquired from the participants using a MAGNETOM ESSENZA device with a power of 1.5 Tesla. This advanced device guarantees high-quality images, allowing for a detailed analysis of the volumes of relevant brain structures by obtaining high-resolution images, thus capturing precise anatomical details of the brain structures. Specific imaging parameters included a slice thickness of 1 mm, a field of view of 256 × 256 mm, and a matrix size of 256 × 256 pixels. These parameters were chosen to optimize the resolution and contrast of the images, enabling accurate volumetric analysis. To perform the MRI volumetry, specific protocols were followed that included conditions, such as the use of a support device to maintain the appropriate position during the procedure. The total time required for image acquisition was adjusted according to the needs of the protocol, thus ensuring complete and accurate collection of the volumetric data. Reproducibility of the volumetric measurements was ensured by performing repeat scans on a subset of participants and comparing the results across different sessions.

The study population made up of 36 individuals who descended from families affected by HD was subjected to this advanced imaging procedure for a detailed analysis of brain volumes, thus providing precise data for the evaluation of possible volumetric changes associated with the premotor phases of HD in this specific population.

##### AI Software for Volumetric Imaging 

AI software (specifically Entelai/IMEXHS, v4.3.4) was used to perform the brain volume assessment from volumetric MRI images. This software is based on the GPT-3 model developed by OpenAI and uses advanced machine learning techniques. The Entelai platform stands out because of its ability to understand and generate text in a coherent and relevant manner in various contexts and applications. The Entelai software (v4.3.4), based on OpenAI’s GPT-3 model, operates using advanced machine learning techniques, specifically using a type of neural network architecture called a “transformer”.

The AI model implemented in Entelai/IMEXHS uses an approach based on deep neural networks to infer brain volumetric changes from magnetic resonance images from the Colombian population allowing it to accurately identify and quantify relevant brain structures in this specific demographic. The inference process includes automatic segmentation of brain areas, followed by a comparative evaluation with previous studies using a linear statistical model that adjusts predictions based on initial volume, patient age, and the time interval between studies. This approach has been shown to be highly accurate, with 95% agreement in detecting significant volumetric changes compared to manual assessments by experienced neuroradiologists [11,28].

The inference process performed by AI revealed that the predicted volumetric changes agreed with the clinical progression observed in premanifest Huntington’s disease patients. AI allowed the identification of changes that, in some cases, anticipated the onset of clinical symptoms by several years, demonstrating its usefulness in early detection and informed clinical decision-making.

In the context of brain volume assessments, Entelai uses these capabilities to interpret data from volumetric MRI images. By understanding the language and instructions related to the specific task, it can perform calculations and analyses to estimate the volumes of the brain structures of interest. Using these programs, accurate calculations of the volumes of various brain structures were performed, including the cortical, subcortical, and superficial regions of the white and gray matter covering both cerebral hemispheres. 

Entelai has been recognized for its ability to process large volumes of medical data efficiently, integrating deep learning algorithms for segmentation and analysis of complex medical images. Its ability to customize analyses according to the clinical context of the patient distinguishes it from other AI platforms that use more generalized methods (https://www.entelai.com, accessed on 15 January 2021) [29]. This platform not only applies already known AI methods, but optimizes and adapts them for more accurate and relevant analysis in the context of neurodegenerative diseases [29,30].

Entelai can significantly contribute to the advancement of early detection of complex diseases. The combination of AI and specialized software allowed us to obtain detailed measurements of brain volumes relevant to studying HD in a Colombian Caribbean population [19,25,26].

### 2.3. Statistical Analysis

Brain volumes were subjected to a normality analysis using the Kolmogorov–Smirnov method. In cases where normality was confirmed, variables were expressed in terms of mean and standard deviation. A comparison between the right and left hemisphere brain volumes was carried out through the Student’s *t*-test.

To evaluate differences between groups, defined by CAG repeat length (≤26 CAG, 27–35 CAG, and ≥40 CAG), a two-way analysis of variance was performed. The Bonferroni test was then applied as a post hoc analysis to identify specific differences between groups.

The Spearman’s test was used to study possible correlations between brain volumes and variables such as age, sex, and disease manifestation status. All analyses were performed using SPSS version 26 software, and graphical representations were created using GraphPad Prism version 10.0. A threshold of statistical significance was set at *p* < 0.05 for all the results obtained in the study.

## 3. Results

The sociodemographic characterization of the study participants is arranged by gender, age, and triplet expansion (CAG) (Table 2). In terms of gender, 67.5% of participants are women, while 32.5% are men. Regarding age, the mean is represented according to the expansion number (34.53 [≤26], 27.5 [25,26,27,28,29,30,31,32,33], and 39.25 [≥40], with the latter being more predominant). As for triplet expansion, 75% of participants had an expansion less than or equal to 26, indicating a normal state, while 20% had an expansion greater than 40, suggesting complete penetrance of the disease. Furthermore, intermediate cases (27–35) were identified in 5% of participants. One case of error (Fail) and two cases with data not available (NA) were recorded. After breaking down the data by gender, women double the population in our study.

The analysis of brain parenchyma volume did not reveal any significant differences across the CAG groups (Kruskal–Wallis H = 4.526, *p* = 0.104). The mean parenchyma volume was 1115.14 cm^3^ in the CAG ≤ 26 group, with no significant differences observed in the CAG 27–35 and CAG ≥ 40 groups.

In contrast, a significant increase in cerebrospinal fluid (CSF) volume was observed in the CAG ≥ 40 group (Kruskal–Wallis H = 13.216, *p* = 0.001), suggesting possible brain atrophy in individuals with higher CAG repeat lengths. The mean CSF volume in the CAG ≤ 26 group was 294.92 cm^3^, with the CAG ≥ 40 group showing the highest mean rank. Regarding gray matter, the analysis showed no significant differences in volume between the groups (Kruskal–Wallis H = 4.366, *p* = 0.113). The CAG ≤ 26 group had a mean volume of 589.78 cm^3^, with similar trends observed across the other groups.

Finally, no significant differences were found in total white matter volume or in the volumes of the left and right hemispheres across the CAG groups (Kruskal–Wallis H values ranging from 2.885 to 4.209, *p* > 0.05). The mean global white matter volume was 525.36 cm^3^ in the CAG ≤ 26 group (Table 3).

**Figure 1:**
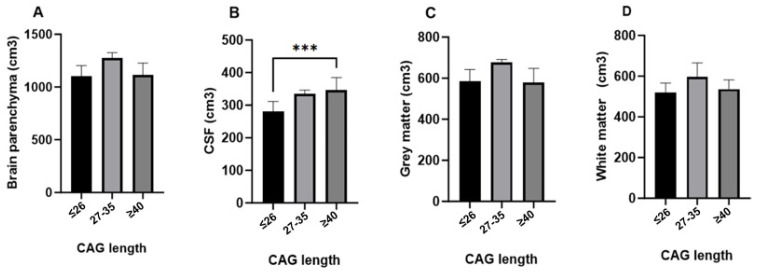
Comparison of global brain volumes according to CAG expansion. This figure compares the global brain volumes in three groups of individuals with different CAG triplet expansions: ≤26 (normal), 27 – 35 (intermediate), and >40 (full penetrance). The structures evaluated include the brain parenchyma volume (**A**), cerebrospinal fluid (CSF) volume (**B**), gray matter volume (**C**), white matter volume (**D**). The *p* values indicate the statistical significance of the observed differences: (*** *p* < 0.00001).

The analysis of brain volumes according to CAG triplet expansion reveals distinct patterns across different brain structures, as illustrated in Figure 1. Brain parenchyma, gray matter, and white matter volumes did not show statistically significant differences between the groups (≤26, 27–35, and ≥40 CAG repeats), although there was a trend of slightly higher volumes in the intermediate group (27–35) compared to the other groups. In contrast, cerebrospinal fluid (CSF) volume showed a significant increase in the full penetrance group (≥40 CAG repeats) compared to the normal repeat group (≤26 CAG repeats), with a highly significant *p* value (*** *p* < 0.00001). This finding suggests that greater CAG repeat lengths are associated with increased CSF volume, potentially reflecting brain atrophy. These results indicate a selective impact of CAG triplet expansion on CSF volume, while other brain structures remain relatively unaffected (Figure 1).

The analysis of subcortical brain structure volumes in relation to CAG triplet expansion reveals significant differences in specific brain regions in individuals at risk of developing Huntington’s disease. Notably, the volumes of the left pallidum (*p* = 0.030) and right pallidum (*p* = 0.018), as well as the left putamen (*p* = 0.011) and right putamen (*p* = 0.007), were significantly reduced in the full penetrance group (≥40 CAG repeats) compared to the other groups, suggesting that a higher number of CAG repeats significantly influences the atrophy of these structures. In contrast, no statistically significant differences were observed in the volumes of the left and right amygdala, left and right caudate, and left and right thalamus (*p* > 0.05), indicating that these structures are not significantly affected by CAG triplet expansion in the premanifest stages of the disease. These findings highlight the selective impact of CAG triplet expansion on subcortical structures, with the pallidum and putamen showing greater susceptibility to early degeneration in Huntington’s disease (Table 4).

**Figure 2:**
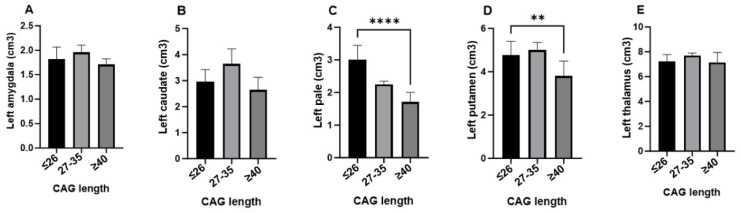

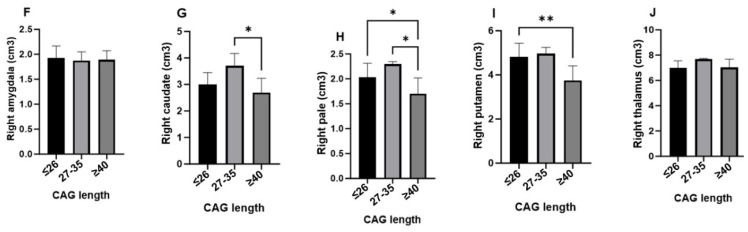
Subcortical structure volumes according to CAG triplet expansion. This figure compares the volumes of various subcortical brain structures in three groups of individuals with different CAG triplet expansions: ≤26 (normal), 27–35 (intermediate), and >40 (full penetrance). The structures evaluated include the amygdala (**A**,**F**), caudate (**B**,**G**), pallidum (**C**,**H**), putamen (**D**,**I**), and thalamus (**E**,**J**), in both the left and right hemispheres. The *p* values indicate the statistical significance of the observed differences: * (*p* < 0.002), ** (*p* < 0.0002), and **** (*p* < 0.000001).

Figure 2 illustrates the impact of CAG triplet expansion on the volumes of subcortical structures in individuals at risk of developing Huntington’s disease, categorized into three groups: ≤26 (normal), 27–35 (intermediate), and >40 (full penetrance). Significant reductions in the volumes of several subcortical structures were observed, particularly in the full penetrance group.

On the left side, subcortical structures, except for the thalamus, showed marked atrophy in the full penetrance group, suggesting a specific vulnerability to CAG triplet expansion in key regions such as the pallidum and putamen. On the right side, significant volume reductions were also observed in the caudate nucleus, globus pallidus, and putamen, consistent with the striatal atrophy characteristic of the disease. However, the right amygdala and thalamus-maintained volumes were comparable to those of the normal and intermediate groups, indicating possible preservation during the early stages of the disease.

These findings highlight the asymmetric and region-specific nature of brain atrophy in Huntington’s disease, with more pronounced effects on subcortical structures involved in motor control and cognitive processes, providing a deeper understanding of neurodegeneration in this disease.

Table 5 highlights the impact of CAG triplet expansion on the volumes of the ventricular system, specifically focusing on the 4th ventricle and supratentorial ventricles in individuals at risk of developing Huntington’s disease. Participants were divided into three groups based on the number of CAG repeats: ≤26 (normal), 27–35 (intermediate), and ≥40 (full penetrance).

For the 4th ventricle, no statistically significant differences in volume were observed across the CAG groups (Kruskal–Wallis H = 3.245, *p* = 0.197), indicating that this structure remains relatively unaffected by CAG repeat expansion. The mean volume for the 4th ventricle was 1.2939 cm^3^ in the normal group, with higher average ranks observed in the intermediate and full penetrance groups.

In contrast, the analysis of the supratentorial ventricles revealed a significant increase in volume in the full penetrance group (Kruskal–Wallis H = 11.765, *p* = 0.003). The mean volume in the c group was 11.0939 cm^3^, with the highest volumes observed in the intermediate and full penetrance groups. This suggests that supratentorial ventricular enlargement is associated with increased CAG repeat lengths, likely reflecting the underlying brain atrophy characteristic of Huntington’s disease.

These findings indicate a differential impact of CAG expansion on the ventricular system, with significant enlargement of the supratentorial ventricles in individuals with higher CAG repeat numbers, while the 4th ventricle remains less affected. This pattern of ventricular dilation aligns with the progressive neurodegeneration seen in Huntington’s disease and may serve as an early marker of disease progression.

**Figure 3:**
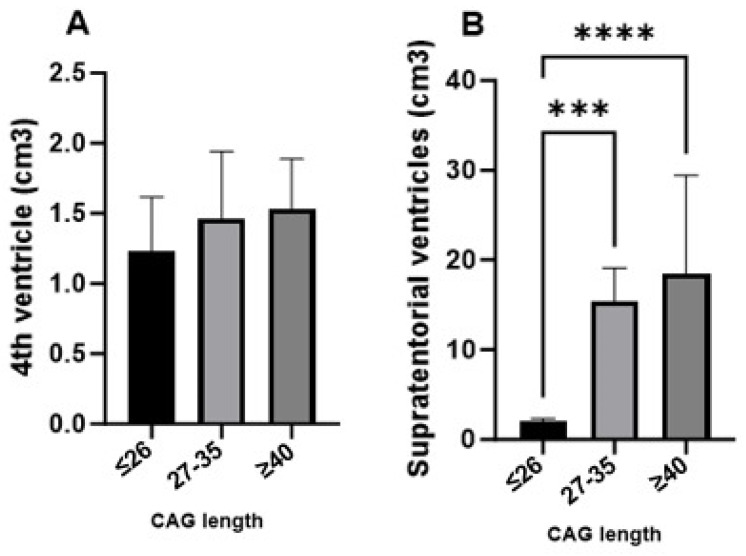
Ventricular system volumes according to CAG triplet expansion. This figure compares the volumes in three groups of individuals with different CAG triplet expansions: ≤26 (normal), 27–35 (intermediate), and >40 (full penetrance). The evaluated structures include the 4th ventricle (**A**) and supratentorial ventricle (**B**). The *p* values indicate the statistical significance of the observed differences: (*** *p* < 0.00001, **** *p* < 0.000001).

Figure 3 compares the volumes of the 4th ventricle (A) and supratentorial ventricles (B) across three groups based on CAG triplet expansion: ≤26 (normal), 27–35 (intermediate), and ≥40 (full penetrance). A significant finding from this analysis is the marked increase in ventricular system volume, both supratentorial and infratentorial, in the full penetrance group. This substantial enlargement, particularly evident in the supratentorial ventricles, is statistically significant, as indicated by the *p* values (*** *p* < 0.00001, **** *p* < 0.000001). These results suggest that individuals with higher CAG repeat lengths experience pronounced ventricular dilation, reflecting the extent of neurodegeneration associated with Huntington’s disease.

The results presented in Table 6 indicate that CAG triplet expansion does not reveal a statistically significant impact on the volumes of infratentorial structures, including cerebellar white and gray matter and the brainstem, in individuals at risk for developing Huntington’s disease. Specifically, variations in mean volumes are observed among three groups (≤26, 27–35, and ≥40 repeats), with those possessing a higher number of repeats showing a trend toward reduced volumes, particularly in cerebellar gray matter. However, none of these differences reach statistical significance, with *p* values exceeding 0.05 in all cases. These findings suggest that while there are fluctuations in the volumes of these brain structures, CAG triplet expansion does not have a significant effect on them within the analyzed cohort.

**Figure 4:**
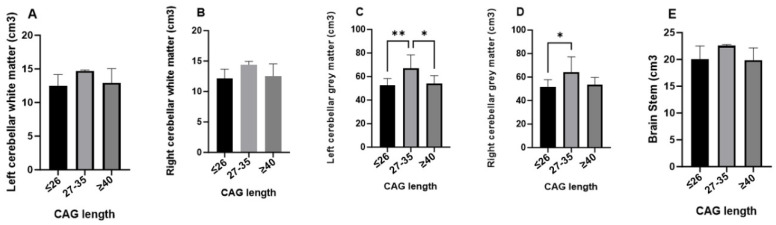
Comparison of infratentorial structure volumes according to CAG triplet expansion. This figure compares the volumes of infratentorial structures in three groups of individuals with different CAG triplet expansions: ≤26 (normal), 27–35 (intermediate), and >40 (full penetrance). The evaluated structures include the left cerebellar white matter volume (**A**), right cerebellar white matter (**B**), left cerebellar gray matter (**C**), right cerebellar, gray matter (**D**), and brainstem (**E**). The *p* values indicate the statistical significance of the observed differences: (* *p* < 0.002), ** *p* < 0.0002).

Figure 4 illustrates the comparison of infratentorial structure volumes across three groups of individuals categorized by CAG triplet expansion: ≤26 (normal), 27–35 (intermediate), and ≥40 (full penetrance). A significant finding from this analysis is the marked increase in left and right cerebellar gray matter in the intermediate group. The statistical significance of the differences observed between the groups is indicated by *p* values, where * *p* < 0.002 and ** *p* < 0.0002, highlighting significant differences in specific infratentorial structures associated with the extent of CAG triplet expansion.

The results presented in Table 7 indicate that CAG triplet expansion does not have a statistically significant impact on the volumes of various cortical areas, including the frontal, insular, occipital, parietal, and temporal cortices, as well as the hippocampus, in individuals at risk of developing Huntington’s disease. Although variations in mean volumes were observed among groups with different numbers of CAG repeats (≤26, 27–35, and ≥40), these differences did not reach statistical significance in any of the regions analyzed. Specifically, in the frontal, parietal, and temporal cortices, as well as in the hippocampal regions, the *p* values exceeded the threshold for significance in all cases, suggesting that, despite fluctuations in volumes, CAG triplet expansion does not have a significant effect on these cortical and hippocampal structures within the studied sample (Table 7).

**Figure 5:**
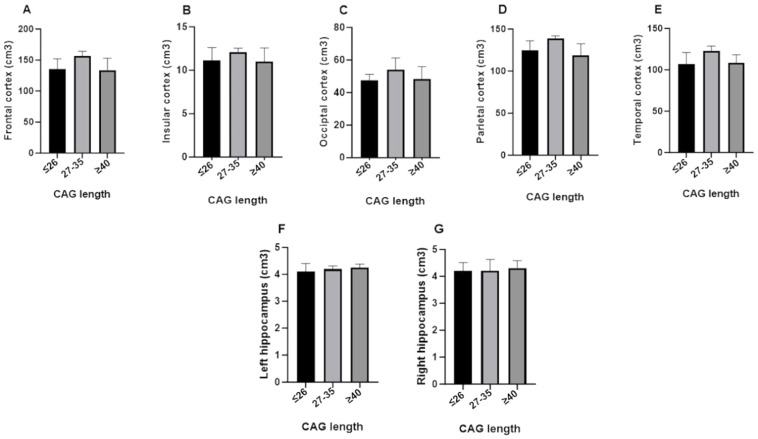
Cortical and hippocampal volumes according to CAG triplet expansion. This figure compares the volumes of various cortical areas and the hippocampus in three groups of individuals with different CAG triplet expansions: ≤26 (normal), 27–35 (intermediate), and >40 (full penetrance). The evaluated structures include the frontal cortex (**A**), insular cortex (**B**), occipital cortex (**C**), parietal cortex (**D**), temporal cortex (**E**), left hippocampus (**F**), and right hippocampus (**G**). The *p* values indicate the statistical significance of the observed differences: (*p* > 0.05).

Figure 5 presents a comparative analysis of the volumes of various cortical areas and the hippocampus in individuals with different CAG triplet expansions, categorized into three groups: normal (≤26 repeats), intermediate (27–35 repeats), and full penetrance (≥40 repeats). The results reveal a significant increase in the volumes of the frontal, insular, occipital, parietal, and temporal cortices in the intermediate group compared to the normal and full penetrance groups. However, no statistically significant differences were observed in the volumes of the hippocampus, both left and right, among the three groups. These findings indicate that while CAG triplet expansion is associated with variations in cortical volumes, the hippocampus remains relatively stable regardless of the number of CAG triplet repeats.

**Scheme 1:**
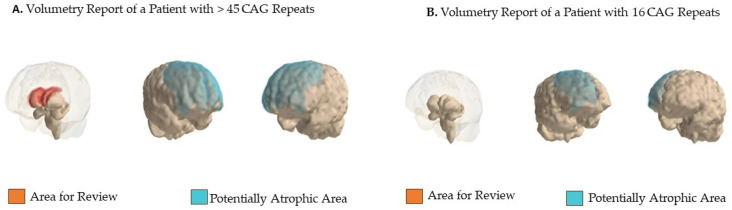
Brain Volumetry via Nuclear Magnetic Resonance Imaging in Individuals with CAG Triplet Expansion.

Scheme 1 displays brain volumetry results obtained through nuclear magnetic resonance imaging in individuals with varying lengths of CAG repeats. Panel A shows the volumetric report of a patient with more than 45 CAG repeats, highlighting potential atrophic areas (indicated in blue) within the cerebral cortex, as well as regions requiring further examination (marked in orange). These results reveal a significant reduction in cortical volume and a more pronounced appearance of atrophic regions compared to Panel B.

Panel B illustrates the volumetric report of a patient with 16 CAG repeats, also identifying potentially atrophic areas (in blue) and zones for additional review (in orange) but to a lesser extent than in the patient with a higher CAG repeat expansion. The difference in the extent of atrophy between the two patients suggests a direct correlation between the number of CAG repeats and the severity of structural brain changes.

These findings support the hypothesis that a higher CAG repeat load is associated with increased cerebral atrophy and variations in brain volume, underscoring the importance of early and detailed evaluations in individuals at high risk for neurodegenerative diseases. Identifying atrophic areas and regions for further review may be crucial for implementing effective intervention and monitoring strategies in disease progression.

## 4. Discussion

This study explores the association between the number of triplet repeats and the volume of various brain structures in premanifest HD patients. This phase is characterized by the absence of notable motor symptoms, although underlying brain changes may already exist, as well as symptoms related to cognitive dysfunction and neuropsychiatric involvement [31]. Early detection of changes in brain structures is essential for developing interventions since certain volumetric changes may precede the manifestation of clinical symptoms [32].

When CSF was assessed, a progressive increase in volume was observed in the intermediate and full penetrance groups, consistent with data on brain atrophy (Figure 3). These findings are more frequent in symptomatic patients and have even been detected up to 10 years before the onset of symptoms [33,34].

With regard to the white matter, the results are consistent with those of previous studies suggesting changes in brain conformation in the premanifest phase and even from childhood [18,32,35,36,37], with a decrease in areas such as the corpus callosum and posterior white matter tracts [18,32,35,36,37]. As the disease progresses to the symptomatic phase, white matter atrophy extends until it becomes global, which has been evidenced in volumetric analyses [38] and in the TRACK-HD and PREDICT-HD reports, which describe the same progression. This is a finding repeated in our study where the full penetrance group shows a decrease in white matter (Figure 4), thus supporting the usefulness of this biomarker to track the progression of the disease. The gray matter volume also shows a decrease in the full penetrance group, which is in line with studies indicating gradual changes in gray matter structures from the premanifest phase to the manifest phase of the disease [39,40]. 

As for the subcortical structures (Figure 2), the striatum is considered the most affected structure in HD, and the underlying mechanisms explaining this regional preference are believed to be related to the instability of the mutation in different tissues. The TRACK-HD report has shown that the caudate volume exceeds that of the putamen at all phases of the disease [11]. However, other studies based on structural resonances have indicated an alteration of 50% in the putamen and 20–30% in the caudate [12]. Our study supports these findings by observing a more significant volume loss in the putamen compared to the caudate. These changes can predict conversion from the presymptomatic to the symptomatic phase in a period of up to 2 years [28].

Regarding the globus pallidus, our results also indicate a decrease in its volume from the premanifest phase, with a notable change on the left side. As for the volume of the thalamus, although the literature emphasizes the significant atrophy in the late stages of HD, in our research, this volume remained relatively similar in the three groups. 

Although not traditionally associated with HD, the amygdala showed differences in volume between groups, especially on the right side compared to the left, in addition to a slight increase in volume in the intermediate group and a slight decrease in the full penetrance group. This highlights the importance of further research in this area.

The ventricular system (Figure 1 and Figure 3), an indicator of total brain atrophy, revealed a progressive increase in its volume as the number of triplets grows, supporting the notion that ventricular dilation reflects the extension of the pathology in extrastriatal regions of the gray and white matter as the disease progresses [41]. Ventricular dilation is seen approximately 5 years before the onset of motor symptoms and approximately a decade after the changes observed in the caudate owing to neuronal loss [42]. Extrastriatal degeneration is also evident in the early stages of the clinical course of HD, which could be considered a global marker of progression, as well as total brain atrophy or ventricular enlargement [43].

Our study found greater involvement of gray matter than cerebellar white matter (Figure 4). Tissue loss of this structure has been reported in juvenile-onset HD and in some cases in adult-onset [44,45]. In most patients, these changes have been reported as moderate.

In studies of cerebellar atrophy, no correlation has been found with CAG repeat [46,47], and there is a nonsignificant trend toward an inverse correlation between cerebellar atrophy and disease duration, given that cerebellar neuronal loss occurs later in the clinical course of the disease [48].

Finally, the brain stem nuclei were affected in HD. This structure shows a generalized neuronal loss with particular involvement of the substantia nigra, the precerebellar pontine nuclei, the inferior olive, the oculomotor reticulotegmental nucleus, the premotor oculomotor area, the interpositus raphe nucleus, the superior auditory olive, and the vestibular nuclei. Patients with HD present autonomic alterations and oculomotor dysfunction, some of which could be explained by brain stem pathology [49]. To date, there is no evidence of brain stem involvement in the premanifest phase of HD. The findings of our study demonstrate that, rather than degeneration or loss of volume of this structure, there is an increase in volume in the intermediate group.

Regarding the brain volumes of cortical areas, both pathological and imaging studies have interpreted that the frontal lobes are altered in HD and that their degree of atrophy would be related to the severity of symptoms and general cognitive function. However, these relationships remained insignificant after considering the total brain volume [41,50]. In other research, individuals with pre-HD presented oculomotor and visuomotor deficits, possibly due to a loss of the visual and prefrontal cortex. In our study, no volumetric difference was observed between patients with normal AGC length and patients with full penetrance, with a paradoxical increase in volume in the intermediate group (Figure 5). 

As for the temporal, occipital, and parietal cortical volumes, it has been found that there may be signs of volume decrease in premanifest phases, but the atrophy itself occurs prior to the onset of symptoms or in the manifest clinical stage of HD, which is directly proportional to the expansion of triplets [47,51]. Despite this, as with the findings in the frontal lobe volume, there was also an increase in volume in the intermediate group, while the volume values between the normal and full penetrance groups were similar.

Structures such as the hippocampus and the insula show abnormal neurodegeneration in premanifest phases. However, the size of the effect is smaller than in the striatal structures, and these changes can occur up to 20 years before clinical diagnosis [38]. In our study, the hippocampus volumes remain similar, except in the insular cortex, where volumes increase in the intermediate group. 

Neurodegeneration in this disease follows a topographic and ordered distribution, starting from the tail of the caudate nucleus to the head and then the body. The most vulnerable neurons in HD are the medium-sized GABAergic spiny projection neurons, which constitute 90–95% of the neuronal population of the striatum, demonstrated by their specific characteristic of bilateral atrophy in this area [52]. Other neuropathological changes occur in different areas of the cerebral cortex (layers III, IV, and V of the prefrontal cortex), which, due to their retrograde degenerative projections, explain the predominance of motor, cognitive, or emotional symptoms in each individual [53].

Macroscopically, the thalamus is usually within normal limits but MRI studies have shown a variable loss in some of its nuclei due to astrocytosis and neuronal loss [54]. The cerebellum, previously considered to be relatively unaffected, now shows a significant loss of Purkinje cells and neurons in the deep cerebellar nuclei [55]. 

The blood–brain barrier is also affected, as aggregates of mutant HTT protein are present in all major components of the neurovascular unit, leading to structural, morphological, and functional changes in cerebral blood vessels [56]. This is where neuroinflammation plays an important role, promoting the removal of cellular waste and acting on neurons, possibly contributing to neuronal death.

Mutant *HTT* is expressed in the glial cells (astrocytes and microglia), thus reducing their neuroprotective function. It also mitigates the production and transport of brain-derived neurotrophic factor in the cortex through the corticostriatal pathway, with dysfunctional cortical signaling [8]. An increase in multiple cytokines has also been observed both in plasma (interleukin [IL]-1b, IL-4, IL-6, IL-8, tumor necrosis factor [TNF]-α, and IL-10) and in the striatum (IL-6, IL-8, and TNF-α), especially IL-6, in carriers of presymptomatic mutations 16 years earlier, which would translate into early inflammatory changes. 

Complement factors C1QC, C2, and C3, as well as other proteins associated with inflammatory pathways, such as peptidoglycan recognition protein 2 and apolipoprotein A4 have been found to increase in the CSF of HD patients compared to controls. The levels of these proteins in CSF followed a trend consistent with increased disease progression [57].

Taken together, these findings enrich the understanding of volumetric variations in the premanifest phase of HD, thus contributing to the identification of potential biomarkers and highlighting the complexity of brain changes associated with triplet expansion. The divergence from the literature in some results underlines the need for additional research to consolidate and contextualize these findings in the context of the clinical heterogeneity of HD since, by the time the clinical stage begins, the disease has progressed markedly, and irreversible lesions are present. Therefore, it would be expected that interventions and treatments during the premanifest stages could be more effective [33]. 

### Limitations

The study presented several limitations, starting with the fact that the sample size was relatively small, 36 individuals from a specific population in the Colombian Caribbean, which could have limited the generalization of the findings to more diverse populations. In addition, selecting the sample among descendants of families affected by HD in a specific geographic location introduced a possible selection bias. The lack of genetic diversity within the sample could also have influenced the applicability of the results to populations with different genetic or ethnic backgrounds. Technical limitations in the acquisition of MRI and the interpretation of AI results should also have been considered. Despite the statistical analyses performed, the results should have been interpreted cautiously, recognizing the possibility of unmeasured variables that could have affected the findings. 

## 5. Conclusions

This descriptive study presents the brain volumetric alterations in individuals with premanifest HD identified using AI software and its correlation with the expansion of triplets in a population from the Colombian coast. 

In the premanifest phase, the increase in volume in the brain parenchyma, CSF, gray and white matter, and ventricular system stands out in the intermediate and full penetrance groups. The cortical and infratentorial areas present greater volume in the intermediate group compared to the normal and full penetrance groups. Regarding the subcortical structures, the striatum shows a significant reduction in the full penetrance group. 

The results obtained suggest that the application of AI in neuroimaging could serve as an alternative to identify structural biomarkers long before patients present clinical symptoms, thus allowing for early interventions and opening the door to future research.

## Figures and Tables

**Table 1:** Inclusion and exclusion criteria for the study.

Inclusion Criteria	Exclusion Criteria
Accepting and signing the informed consent form	Not accepting and signing the informed consent form
Belonging to a family with a record of at least one member with Huntington’s disease	Not being affiliated to the General Health Social Security System
Confirming the anomalous expansion of cytosine, adenine, and guanine triplets in the *IT15* gene	Patients with movement disorders and/or a history of psychiatric disorders other than Huntington’s disease
Being affiliated to the General Health Social Security System	

**Table 2:** Demographic profile of study participants.

Variables	Triplet Expansion ≤26	Triplet Expansion 27–35	Triplet Expansion ≥40	Fail/NA	Total
**Age**	$\bar{x}$ 34.53 (σ10.40)	$\bar{x}$ 27.50 (σ10.60)	$\bar{x}$ 39.25 (σ12.44)	-	-
Gender					
Female (26)	18	1	4	3	26
Male (13)	10	1	2	0	13

Note: $\bar{x}$ represents the mean age in years. The percentages in parentheses indicate the proportion of the population within each triplet expansion category. “Triplet expansion ≤ 26” refers to individuals with a triplet repeat length of 26 or fewer. “Triplet expansion 27–35” refers to individuals with a triplet repeat length between 27 and 35. “Triplet expansion ≥ 40” refers to individuals with a triplet repeat length of 40 or more. “Fail/NA” indicates data that were not available or cases where relevant information could not be obtained. The “Total” column represents the sum of all individuals across all categories.

**Table 3:** Influence of CAG triplet expansion on brain substructure volumes: This table illustrates how CAG triplet expansion affects the volumes of various brain substructures in individuals at risk of developing Huntington’s disease. The evaluated structures include brain parenchyma, cerebrospinal fluid (CSF), gray matter, white matter, and white matter in the left and right hemispheres. Participants were categorized based on the number of CAG repeats: ≤26 (normal), 27–35 (intermediate), and ≥40 (full penetrance).

Structures	Substructures	CAG	N	Average Range	Mean	Standard. Deviation	Minimum	Maximum	Kruskal–Wallis’s H Test	*p*
Global volumes	Brain parenchyma	≤26	28	17.21	1115.14	105.204	918	1350	4.526	0.104
		27–35	2	33.50						
		≥40	6	19.50						
	CSF	≤26	28	15.09	294.92	40.353	230	411	**13.216**	**0.001**
		27–35	2	30.00						
		≥40	6	30.58						
	Gray matter	≤26	28	17.39	589.78	59.892	456	722	4.366	0.113
		27–35	2	33.50						
		≥40	6	18.67						
	White matter	≤26	28	16.98	525.36	50.749	445	645	3.773	0.152
		27–35	2	30.75						
		≥40	6	21.50						
	White matter, left hemisphere	≤26	28	16.84	239.64	23.411	202	295	4.209	0.122
		27–35	2	31.00						
		≥40	6	22.08						
	White matter, right hemisphere	≤26	28	17.18	240.39	23.369	204	298	2.885	0.236
		27–35	2	29.25						
		≥40	6	21.08						

Note: For the analysis of brain volumes and triplet expansion, the following statistical tests were used: Kolmogorov–Smirnov method, Student’s *t*-test, two-way analysis of variance with Bonferroni post hoc test, and Spearman’s test. The *p* value of 0.001 is shown in black. Source: own elaboration.

**Table 4:** Influence of CAG triplet expansion on subcortical brain structure volumes. This table illustrates how CAG triplet expansion affects the volumes of various subcortical brain structures in individuals at risk of developing Huntington’s disease. The evaluated structures include the left and right amygdala, caudate, pallidum, putamen, and thalamus. Participants were categorized based on the number of CAG repeats: ≤26 (normal), 27–35 (intermediate), and ≥40 (full penetrance).

Structures	Substructures	CAG	N	Average Range	Mean	Standard Deviation	Minimum	Maximum	Kruskal–Wallis H Test	*p*
Subcortical structures	Left amygdala	≤26	28	18.91	1.8069	0.23091	1.40	2.41	2.811	0.245
		27–35	2	27.50						
		≥40	6	13.58						
	Right amygdala	≤26	28	18.86	1.9189	0.22147	1.56	2.38	0.158	0.924
		27–35	2	18.00						
		≥40	6	17.00						
	Left caudate	≤26	28	18.98	2.9500	0.49608	1.69	4.05	5.449	0.066
		27–35	2	31.50						
		≥40	6	11.92						
	Right caudate	≤26	28	18.89	2.9908	0.49358	1.78	4.03	5.494	0.064
		27–35	2	32.00						
		≥40	6	12.17						
	Left pallidum	≤26	28	19.52	1.9750	0.30807	1.30	2.64	**6.998**	**0.030**
		27–35	2	30.50						
		≥40	6	9.75						
	Right pallidum	≤26	28	19.50	1.9883	0.31310	1.19	2.65	**8.082**	**0.018**
		27–35	2	32.00						
		≥40	6	9.33						
	Left putamen	≤26	28	20.52	4.6286	0.71912	2.85	5.64	**9.043**	**0.011**
		27–35	2	25.00						
		≥40	6	6.92						
	Right putamen	≤26	28	20.73	4.6425	0.72777	2.78	5.66	**9.915**	**0.007**
		27–35	2	24.00						
		≥40	6	6.25						
	Left thalamus	≤26	28	17.71	7.2292	0.59440	5.73	8.60	1.964	0.375
		27–35	2	28.50						
		≥40	6	18.83						
	Right thalamus	≤26	28	17.41	7.0389	0.57174	5.92	8.27	3.376	0.185
		27–35	2	31.50						
		≥40	6	19.25						

Note: For the analysis of brain volumes and triplet expansion, the following statistical tests were used: Kolmogorov–Smirnov method, Student’s *t*-test, two-way analysis of variance with Bonferroni post hoc test, and Spearman’s test. The *p* values of 0.030, 0.018, 0.011, and 0.007 are shown in black. Source: own elaboration.

**Table 5:** Impact of CAG expansion on ventricular volumes. This table illustrates the impact of CAG triplet expansion on the volumes of the ventricular system, including the supratentorial ventricles and 4th ventricle, in individuals at risk of developing Huntington’s disease. The participants are categorized based on the number of CAG repeats: ≤26 (normal), 27–35 (intermediate), and ≥40 (full penetrance).

Structures	Substructures	CAG	N	Average Range	Mean	Standard Deviation	Mini- mum	Maxi- mum	Kruskal–Wallis H Test	*p*
Ventricular system	4th ventricle	≤26	28	16.82	1.2939	0.39211	0.60	2.07	3.245	0.197
		27–35	2	23.00						
		≥40	6	24.83						
	Supratentorial ventricles	≤26	28	15.29	11.0939	6.48371	3.73	39.10	**11.765**	**0.003**
		27–35	2	31.00						
		≥40	6	29.33						

Note: For the analysis of brain volumes and triplet expansion, the following statistical tests were used: Kolmogorov–Smirnov method, Student’s *t*-test, two-way analysis of variance with Bonferroni post hoc test, and Spearman’s test. The *p* values of 0.003 are shown in black. Source: own elaboration.

**Table 6:** Impact of CAG expansion on infratentorial structure volumes: This table illustrates the impact of CAG triplet expansion on the volumes of infratentorial structures, including the left/ right cerebellar white/gray matter and brainstem, in individuals at risk of developing Huntington’s disease. The participants are categorized based on the number of CAG repeats: ≤26 (normal), 27–35 (intermediate), and >40 (full penetrance).

Structures	Substructures	CAG	N	Average Range	Mean	Standard Deviation	Minimum	Maximum	Kruskal–Wallis H Test	*p*
Infratentorial structures	White matter, left cerebellum	≤26	28	17.04	12.7117	1.72630	8.92	15.60	4.551	0.103
		27–35	2	33.00						
		≥40	6	20.50						
	White matter, right cerebellum	≤26	28	16.95	12.3500	1.61168	8.74	15.30	4.607	0.100
		27–35	2	32.75						
		≥40	6	21.00						
	Gray matter, left cerebellum	≤26	28	17.00	53.575	6.9595	42.2	75.1	4.397	0.111
		27–35	2	32.50						
		≥40	6	20.83						
	Gray matter, right cerebellum	≤26	28	16.96	52.783	6.8373	42.3	73.4	3.732	0.155
		27–35	2	30.50						
		≥40	6	21.67						
	Brainstem	≤26	28	17.79	20.197	2.3384	15.6	24.9	2.421	0.298
		27–35	2	29.75						
		≥40	6	18.08						

Note: For the analysis of brain volumes and triplet expansion, the following statistical tests were used: Kolmogorov–Smirnov method, Student’s *t*-test, two-way analysis of variance with Bonferroni post hoc test, and Spearman’s test. The *p* values show no statistically significant differences. Source: own elaboration.

**Table 7:** Impact of CAG expansion on cortical and hippocampal volumes. This table illustrates the impact of CAG triplet expansion on the volumes of various cortical areas, including the frontal, insular, occipital, parietal, and temporal cortices, as well as the hippocampus, in individuals at risk of developing Huntington’s disease. The participants are categorized based on the number of CAG repeats: ≤26 (normal), 27–35 (intermediate), and ≥40 (full penetrance).

Structures	Substructures	CAG	N	Average Range	Mean	Standard Deviation	Minimum	Maximum	Kruskal–Wallis H Test	*p*
Cortical areas	Frontal cortex	≤26	28	17.66	136.736	16.6920	99.5	178.0	3.484	0.175
		27–35	2	32.00						
		≥40	6	17.92						
	Left frontal cortex	≤26	28	17.79	68.853	8.4647	50.2	88.8	3.228	0.199
		27–35	2	31.50						
		≥40	6	17.50						
	Right frontal cortex	≤26	28	17.48	68.164	8.3325	49.3	89.3	4.327	0.115
		27–35	2	33.50						
		≥40	6	18.25						
	Insular cortex	≤26	28	18.04	11.1517	1.45717	8.42	14.50	1.222	0.543
		27–35	2	26.50						
		≥40	6	18.00						
	Left insular cortex	≤26	28	18.11	5.5375	0.75031	4.11	7.17	1.572	0.456
		27–35	2	27.50						
		≥40	6	17.33						
	Right insular cortex	≤26	28	17.73	5.6142	0.71584	4.30	7.35	1.481	0.477
		27–35	2	27.00						
		≥40	6	19.25						
	Occipital cortex	≤26	28	17.07	48.203	4.6208	34.8	59.3	2.981	0.225
		27–35	2	28.75						
		≥40	6	21.75						
	Left occipital cortex	≤26	28	17.52	23.686	2.3424	17.3	28.2	1.982	0.371
		27–35	2	28.00						
		≥40	6	19.92						
	Right occipital cortex	≤26	28	16.75	24.511	2.5215	17.5	31.1	4.534	0.104
		27–35	2	31.25						
		≥40	6	22.42						
	Parietal cortex	≤26	28	18.41	124.433	11.9950	95.6	152.0	4.523	0.104
		27–35	2	32.50						
		≥40	6	14.25						
	Left parietal cortex	≤26	28	18.36	61.4975	5.90719	48.00	76.20	4.085	0.130
		27–35	2	32.00						
		≥40	6	14.67						
	Right parietal cortex	≤26	28	18.39	62.892	6.2177	47.7	76.2	5.154	0.076
		27–35	2	33.50						
		≥40	6	14.00						
	Temporal cortex	≤26	28	17.63	108.156	13.1853	84.8	137.0	3.244	0.197
		27–35	2	31.50						
		≥40	6	18.25						
	Left temporal cortex	≤26	28	17.46	55.164	6.6123	43.5	69.9	3.330	0.189
		27–35	2	31.50						
		≥40	6	19.00						
	Right temporal cortex	≤26	28	17.61	52.906	6.6293	41.3	66.9	3.248	0.197
		27–35	2	31.50						
		≥40	6	18.33						
	Left hippocampus	≤26	28	17.23	4.1364	0.27691	3.58	4.69	1.897	0.387
		27–35	2	21.25						
		≥40	6	23.50						
	Right hippocampus	≤26	28	18.00	4.2328	0.29162	3.74	4.88	0.622	0.733
		27–35	2	16.50						
		≥40	6	21.50						

Note: For the analysis of brain volumes and triplet expansion, the following statistical tests were used: Kolmogorov–Smirnov method, Student’s *t*-test, two-way analysis of variance with Bonferroni post hoc test, and Spearman’s test. The *p* values show no statistically significant differences. Source: own elaboration.

## Data Availability

The data presented in this study are available upon reasonable request from the corresponding authors. They are not publicly available due to the ongoing nature of the study and our commitment to protecting the privacy and confidentiality of our patients.

## Abbreviations

HD	Huntington’s disease
*HTT*	Huntingtin gene (protein coding)
CAG	Cytosine, adenine, and guanine
VBM	Voxel-based morphometry
AI	Artificial intelligence
